# Acute Effects of a Mini-Trampoline Training Session for Improving Normalized Symmetry Index in Participants with Higher Baseline Inter-Limb Asymmetry

**DOI:** 10.3390/healthcare14020160

**Published:** 2026-01-08

**Authors:** Olga Papale, Emanuel Festino, Marianna De Maio, Francesca Di Rocco, Silvia Zema, Cristina Cortis, Andrea Fusco

**Affiliations:** 1 Department of Human Sciences, Society and Health, University of Cassino and Lazio Meridionale, 03043 Cassino, Italy; olga.papale@unicas.it (O.P.); emanuel.festino@unicas.it (E.F.); marianna.demaio@unicas.it (M.D.M.); francesca.dirocco@uniroma5.it (F.D.R.); silvia.zema@unicas.it (S.Z.); 2 European University of Technology EUt+, 03043 Cassino, Italy; 3 Department of Human Sciences and Promotion of the Quality of Life, “San Raffaele” Open University of Rome, 00166 Rome, Italy; 4 Department of Medicine and Aging Sciences, University “G. d’Annunzio” of Chieti-Pescara, 66100 Chieti, Italy; andrea.fusco@unich.it

**Keywords:** prevention, inter-limb asymmetry, health promotion, countermovement jump, plyometric

## Abstract

**Highlights:**

**What are the main findings?**

- A single mini-trampoline training session decreased inter-limb asymmetry in participants with higher baseline inter-limb asymmetry, without affecting countermovement jump performance.
- Participants with lower baseline inter-limb asymmetry did not show a reduction in inter-limb asymmetry, showing a decrease in CMJ performance, possibly reflecting fatigue-related compensatory strategies.

**What are the implications of the main findings?**

- Mini-trampoline training may serve as an effective exercise to acutely reduce asymmetries in individuals at higher injury risk.
- Given its low impact, mini-trampoline training represents a safe and accessible plyometric exercise to use in preventive and rehabilitative programs.

**Abstract:**

**Background**: Inter-limb asymmetry has implications for both athletic performance and healthcare practice. High baseline inter-limb asymmetries have been associated with impaired mobility, increased fall risk, and musculoskeletal injuries across the lifespan. Exercise interventions able to stimulate the stretch–shortening cycle (e.g., plyometric training and jump training) have been shown to have a good impact on asymmetries. Among these, Mini-Trampoline Training (MTT) has recently emerged as potentially effective in reducing asymmetries. **Objectives**: The study aimed to evaluate the acute effects of a single MTT session on muscle power and inter-limb asymmetry in young adults. **Methods**: Twenty-eight recreationally active participants (25.6 ± 2.4 years) completed one MTT session. Before (PRE) and after (POST) the MTT session, single-leg 6 m Timed Hop (6MTH) and countermovement jump (CMJ) tests were administered. Additionally, 6MTH values of the dominant (DOM) and non-dominant (NODOM) limbs were used to stratify participants according to higher (HBIA) or lower (LBIA) baseline inter-limb asymmetry, based on a commonly adopted Normalized Symmetry Index (NSI) threshold (NSI ≥ 10%, n = 12; NSI < 10%, n = 16). Repeated-measures mixed models were used to evaluate the effects of the MTT session on 6MTH, NSI, and CMJ. **Results:** Regardless of group and limb, significant (*p* < 0.0001) improvements in 6MTH (PRE: 2.5 ± 0.06 s; POST: 2.3 ± 0.05 s) were found. Interestingly, the MTT session had a significant (*p* = 0.01) effect on both groups, with a significant (*p* = 0.003) interaction with NSI values, showing an improvement for HBIA (PRE = 15.4 ± 1.1%, POST = 11.3 ± 2.1%), whereas a decrement in LBIA was recorded (PRE = 5.1 ± 0.6%, POST = 9.6 ± 1.5%). CMJ did not show any changes in HBIA (PRE: 36.2 ± 0.9 cm; POST: 35.1 ± 0.7 cm), while a significant (*p* = 0.007) decrease was found in LBIA (PRE: 34.8 ± 1.2 cm; POST: 33.2 ± 1.3 cm). **Conclusions**: A single MTT session induced acute neuromuscular fatigue, reflected by reduced CMJ performance and improved (~8%) inter-limb control during hopping. The HBIA group preserved jump height (~36 cm) and demonstrated a significant reduction in asymmetry (NSI: −4.1%), suggesting more balanced lower-limb recruitment. Conversely, LBIA showed a significant decrease in CMJ and an increased NSI (+4.5%), possibly reflecting fatigue-related compensatory strategies. Overall, a single MTT elicited distinct responses according to baseline asymmetry, supporting its potential as an adaptable modality for enhancing neuromuscular function in HBIA.

## 1. Introduction

Inter-limb asymmetry, or inter-limb difference, referring to the differences in function or performance between upper or lower limbs [1], is a relevant parameter in both athletic performance monitoring and healthcare practice [2]. Several methodologies to quantify asymmetries have been established as the difference between dominant and non-dominant [3], stronger and weaker [4], right and left [5] and injured and un-injured [6] limbs. Despite this variety of methodologies, no standardized method has been established, with the percentage difference between limbs remaining the most widely used approach [7].

Evidence [8,9,10,11] indicates that inter-limb asymmetry is prevalent among adolescents and elite athletes engaged in various sports (e.g., football, short track speed skating, volleyball, basketball, and karate) as a consequence of repeated unilateral or sport-specific motor patterns. A similar phenomenon is observed in non-athletic populations, such as older adults, where inter-limb asymmetry occurs due to age-related neuromuscular decline, sarcopenia, or neurological conditions (e.g., Parkinson’s disease and Alzheimer’s disease) [12]. Moreover, high baseline inter-limb asymmetry levels (≥10–15%) can compromise gait efficiency and increase fall risk [5,13]. Given their implications across both performance- and health-related domains, there is growing interest in interventions aimed at treating both participants with higher and lower baseline inter-limb asymmetries within sports and healthcare contexts.

The most effective non-pharmacological strategies for the treatment of inter-limb asymmetry are found in exercise-based interventions [14]. In particular, plyometric exercises have been shown to enhance neuromuscular function, improving performance and decreasing injury risk in both athletic and participants with higher baseline inter-limb asymmetry populations, through improvements in stretch–shortening cycle function, muscular strength, power, and coordination [14,15]. Among these activities, mini-trampoline training (MTT) represents a form of plyometric exercise combining aerobic and anaerobic demands with rhythmic and music-driven movements. In particular, MTT has been shown to improve bone metabolism, body composition, balance, and neuromuscular coordination in different populations [16,17,18]. By reducing impact forces compared with traditional plyometric drills performed on rigid surfaces, MTT offers a safer and more accessible training modality. In addition, the compliant surface of the mini-trampoline allows the execution of repetitive plyometric actions with lower joint loading and mechanical stress, which may be particularly suitable for participants with higher baseline inter-limb asymmetry, balance deficits, or increased injury risk. These characteristics make MTT a promising alternative to traditional plyometric training in healthcare and preventive contexts. Despite its high potential, little is known about the acute effects of MTT on unilateral and bilateral functional lower-limb tasks.

Therefore, the present study aimed to examine the acute effects of a single MTT session on unilateral and bilateral jumping performance and inter-limb asymmetry in young adults with higher and lower baseline inter-limb asymmetry. We hypothesized that a single MTT session would acutely reduce asymmetry in participants with higher baseline inter-limb asymmetry, while not inducing clinically relevant increases in asymmetry in participants with lower baseline inter-limb asymmetry, thereby supporting its potential application in both health-related and injury-prevention contexts.

## 2. Materials and Methods

### 2.1. Experimental Approach to the Problem

This observational cohort study was conducted in accordance with the Declaration of Helsinki and the study protocol was approved on 4 December 2019 by the Institutional Review Board of the Department of Human Sciences, Society, and Health of the University of Cassino and Lazio Meridionale (approval number 26898) to evaluate the effects of the MTT on participants’ Normalized Symmetry Index (NSI) and unilateral and bilateral jumping performance.

The 6 m Timed Hop (6MTH) test is widely adopted for the assessment of unilateral lower-limb performance and inter-limb asymmetry [19]. Given that both MTT and the 6MTH test involve repeated plyometric actions, examining the effects of MTT on 6MTH performance may help clarify its acute influence on neuromuscular function and asymmetry. Traditionally, inter-limb asymmetry in the 6MTH has been quantified using the Ratio Index, Symmetry Index, Symmetry Angle, or Gait Asymmetry [20]. However, these indices may produce non-linear results and perform asymptotically as values increase and do not provide a clear upper limit for asymmetry, which makes it difficult to interpret what should be considered a meaningful maximum value [20]. The NSI addresses these limitations by providing linear values, being normalized to variable magnitude and functioning as a bounded symmetry index. In the present study, the baseline NSI values were used to classify participants as those with higher baseline inter-limb asymmetry or lower baseline inter-limb asymmetry. Considering that MTT involves dynamic, elastic, and plyometric components relevant to lower-limb function and injury prevention, it could be interesting, in health contexts, to evaluate whether dynamic inter-limb asymmetries are influenced by a single session of MTT. Moreover, given that individuals with different baseline inter-limb asymmetry levels may respond differently to the training, this study also investigated the acute effects on unilateral hopping and vertical jump performance in individuals with higher and lower baseline inter-limb asymmetry.

To complement unilateral assessments, explosive performance was also evaluated through the Countermovement Jump (CMJ), a widely used measure of lower-limb power. The CMJ was included to provide additional information on global neuromuscular responses to MTT, particularly with respect to acute neuromuscular fatigue, given its sensitivity to short-term neuromuscular impairment [21,22]. Although the CMJ is performed bilaterally and does not directly quantify inter-limb asymmetry, its jump height reflects the combined force and power output of both limbs and is sensitive to acute neuromuscular fatigue [23]. In this context, CMJ performance was used to help interpret whether changes observed in unilateral hopping and asymmetry indices occurred alongside signs of generalized fatigue or altered neuromuscular function following MTT.

The assessment of bilateral and unilateral jumping performance was completed before (PRE) and after (POST) MTT training. Baseline NSI values obtained at PRE were used exclusively for participant classification into higher (≥10%) and lower (<10%) baseline inter-limb asymmetry. Changes in NSI following the MTT session (POST–PRE) were then analyzed as outcome measures to evaluate acute modifications in inter-limb asymmetry. Thus, group classification and outcome assessment were based on temporally distinct measurements. In the absence of a non-exercise control condition, participants with lower baseline inter-limb asymmetry were included as a reference comparison group, allowing the examination of whether acute responses to MTT differed according to baseline asymmetry levels. This comparative approach was intended to distinguish changes potentially related to the training stimulus from those attributable to repeated testing or time-related effects, while acknowledging the observational nature of the study design. Additionally, to avoid the influence of fatigue on the evaluation, participants were asked to refrain from moderate-to-vigorous physical activity for 24 h before the experimental sessions.

### 2.2. Participants

Thirty-two (17 females and 15 males) recreationally active students voluntarily participated in this study and received information about the procedures and the aim of the study. All participants were asked to read and sign the consent form, and they were able to withdraw from the study at any time for any reason, without any consequences. Participants with prior experience in MTT and those who reported preexisting limitations such as cardiovascular, respiratory, metabolic diseases, and musculoskeletal injuries of the back or lower extremities were excluded from the study. Participants were classified as recreationally active and included in the study if they reported participating in at least 150 to 300 min of moderate-intensity exercise or 75 to 150 min of vigorous-intensity exercise per week, plus muscle-strengthening activities 2 or more days per week, over the previous 6 months [24,25]. These criteria were used to exclude sedentary individuals and frequent MTT users and were not intended to provide a detailed quantification of habitual physical activity levels or exercise intensity. In accordance with Regulation (EU) 2016/679, to maintain anonymity, each participant was assigned a unique identification code, and personal data were solely used for statistical purposes.

### 2.3. Procedures

Before starting the testing session, individuals’ anthropometric measures were collected. Height and body mass were measured to the nearest 0.1 cm and 0.1 kg, respectively, by using a scale with an integrated stadiometer (Seca 709, Vogel & Halke, Hamburg, Germany). Body mass index (BMI) was subsequently calculated and reported as weight (kg)/height (m^2^). Preferred dominant lower limb was determined using a functional task, defined as the limb spontaneously used when stepping on a platform, in accordance with the previous literature [18,26].

### 2.4. Mini-Trampoline Training

MTT session was performed on a mini-trampoline (CoalSport, Rome, Italy) with the diameter of the elastic surface of 122 cm. The MTT included a session of free practice on the mini-trampoline and 30 min of workout. During the warm-up (5 min) and cool down (5 min) phases, participants were required to continuously jump on the mini-trampoline while performing breathing and mobility exercises. The central phase (20 min) consisted of jumping exercises, including single and double hops on different planes, and jumping jacks, double hops with wide and close stances, performed in combination with movements of the upper body. The total duration for the session was ~40 min [27].

### 2.5. 6 m Timed Hop

The 6MTH test involved multiple consecutive single limb hops on the same limb over a distance of 6 m. A 6 m long, 15 cm wide line was marked on the floor with a standard tape measure placed along the middle, perpendicular to the starting line. Before the tests, participants performed a standardized warm-up of 5 min at low resistance on an upright bike at a self-determined intensity, followed by mobility exercise for the lower limb. After warm-up, all participants completed a familiarization phase of 3 practice trials of 6MTH, to ensure correct understanding of the test procedures and reduce potential learning effects [24]. Following the practice trials, participants performed one testing trial of the 6MTH per limb. Participants stood on one limb (dominant or non-dominant) with their toes aligned at the starting line and began hopping upon the verbal ‘3-2-1’ countdown given by the principal investigator. Participants were instructed to hop over the designated distance in as little time as possible and then gradually slow down to a stop, after which they were asked to walk back to the starting line at a comfortable pace. The test ended when the heel of the tested limb crossed the finish line. Both limbs were tested in random order, and no restrictions were given to participants regarding arm movement. A rest period of 30 s was given between limbs. The time taken to cross the 6 m distance was recorded in seconds using a handheld stopwatch (Seiko Stopwatch, Soler Standard SVAJ001) capable of measuring to the 1/100th of a second and recording multiple split times. The stopwatch was operated by a certified Italian Timekeepers Federation operator, trained in manual timing procedures and movement analysis techniques. Measurements were recorded to the nearest 100th of a second.

### 2.6. Normalized Symmetry Index

The NSI was used to assess symmetry between the dominant and non-dominant limb, calculated as follows [20]:

(1) $$\frac{\left(ndl-dl\right)}{max=\left(0,ndl,dl\right)-min=(0,ndl,dl)}\cdot 100$$

where ndl = non-dominant limb, dl = dominant limb, max = highest value between non-dominant and dominant, and min = lowest value between non-dominant and dominant.

In this formula, the numerator represents the difference between the non-dominant and dominant limb. The denominator represents the maximum and minimum values for the measure across the trial. Given that the values of 6MTH performance are positive, 0 was used as the minimum value. The NSI provides a normalized percentage of asymmetry, where a value of 0% indicates perfect symmetry and a maximum value of 100% indicates maximum asymmetry between limbs. As proposed by previous studies [28,29], an asymmetry threshold of ≥10%, indicative of a higher risk of injury, was used to classify participants as having higher baseline inter-limb asymmetry.

**Figure 1:**
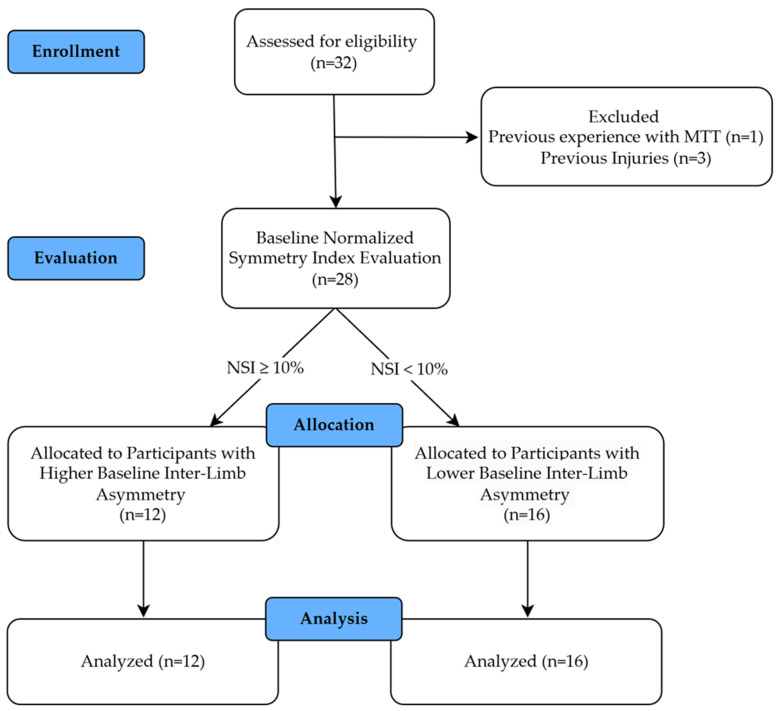
Flow chart of the recruitment and selection process of the subjects included in the study. MTT: Mini-Trampoline Training; NSI: Normalized Symmetry Index.

From a total of thirty-two participants who began the study, four were excluded due to not meeting the inclusion criteria. Subsequently, the twenty-eight subjects were allocated into two groups, according to their baseline asymmetry values. The number of participants with higher baseline inter-limb asymmetry was 12, while the number of participants with lower baseline inter-limb asymmetry was 16 (Figure 1). All participants were healthy young adults, with the ≥10% NSI threshold being used exclusively for baseline stratification purposes and not indicating a clinical diagnosis.

### 2.7. Countermovement Jump

Participants were instructed to stand with their feet shoulder-width apart to perform a fast downward movement to approximately 90° of flexion at the hip and knee, immediately followed by an upward movement. Participants were asked to jump as high as possible, keeping their hands on their hips, to make their trunk as upright as possible throughout the entire jump, and land with their legs fully extended. To familiarize the participants with the jumping technique, a number (3 to 5) of submaximal jumps were conducted before data collection. Subsequently, participants performed three jumps, with a 1 min recovery between attempts, and the best was used for analysis. This approach was adopted to reduce the potential impact of confounding factors such as neuromuscular fatigue and fluctuations in performance related to circadian rhythms, both of which can affect motor output and measurement reliability. CMJ performance was recorded using the Quattro Jump 9290BA force plate (Kistler, Winterthur, Switzerland). Data were recorded at a sampling frequency of 500 Hz. The force plate calculated the time between the take-off and landing phases, used to establish jump heights [30].

### 2.8. Statistical Analysis

STATA software version 18 (StataCorp, College Station, TX, USA) was used for statistical analysis. Means, standard deviations (SD) and 95% confidence intervals (95% CI) were calculated for all variables. The Shapiro–Wilk test was used to assess the normal distribution of the data. Linear repeated-measures mixed model analyses were performed to examine the effects of the MTT on individuals’ 6MTH, NSI, and CMJ performance. Linear repeated-measures mixed model analyses were intentionally chosen because they account for individual variability and are more robust to violations of sphericity [31]. Effect Size (*d*, $\eta_{p}^{2}$) was calculated to determine the magnitude of the effects. Cohen’s *d* was interpreted as trivial (<0.2), small (0.2 to 0.6), moderate (0.61 to 1.2), or large (>1.2), and $\eta_{p}^{2}$ was interpreted as 0.01 (small), 0.06 (medium), and 0.14 (large) [32]. For these analyses, the level of significance was set at *p* < 0.05. Because baseline NSI was used for stratification, inferential interpretation focused on PRE–POST changes in NSI and the time × baseline asymmetry interaction, rather than on baseline group differences, which were expected by design. Moreover, because the asymmetry classification (≥10% NSI) was derived from baseline NSI values and could be influenced by regression to the mean, a sensitivity analysis was performed to avoid dichotomization. Specifically, baseline NSI (PRE) was calculated for each participant and included as a continuous moderator in a repeated-measures mixed-effects model testing the time × baseline NSI interaction (PRE vs. POST). Model estimation was performed using restricted maximum likelihood with the subject specified as a random effect.

## 3. Results

The anthropometric characteristics of the participants, divided by baseline NSI values, are presented in Table 1.

The analysis showed a significant main effect of time (F_(1,83)_ = 5.82, *p* = 0.01, $\eta_{p}^{2}$ = 0.09), indicating a difference in 6MTH performance between PRE (*d* = 0.21; dominant: 2.46 ± 0.48 s; non-dominant: 2.51 ± 0.41 s) and POST (*d* = 0.27; dominant: 2.36 ± 0.44 s; non-dominant: 2.38 ± 0.44 s) MTT session regardless of group and limb (Supplementary Figure S1).

The model was found to be statistically significant for NSI (Supplementary Figure S2; F_(1,26)_ = 8.94, *p* = 0.0003), with a significant main effect found for time (*p* < 0.001, 95%CI: 6.21 to 14.51) and group (*p* = 0.01, 95%CI: 1.09 to 8.23), and a significant interaction (*p* = 0.003, 95%CI: −14.11 to −3.21). Specifically, NSI values were different between PRE and POST in both participants with higher baseline inter-limb asymmetry (*d* = 0.69; PRE: 15.4 ± 3.8%; POST: 11.3 ± 7.4%) and participants with lower baseline inter-limb asymmetry (*d* = 0.99; PRE: 5.0 ± 2.7%; POST: 9.7 ± 6.1%) populations. Additionally, PRE values were significantly different among groups (*d* = 3.1; *p* < 0.001, 95%CI: 6.39 to 14.32); thus, the difference did not persist in POST evaluation (*p* = 0.4, 95%CI: −2.26 to 5.65). Additionally, a significant time × baseline NSI interaction was observed (β = −0.78, SE = 0.21, *p* < 0.001), indicating that both the magnitude and the direction of PRE–POST changes in asymmetry depended on baseline NSI values.

Lastly, CMJ analysis showed the significance of the model (F_(3,26)_ = 3.39, *p* = 0.003, $\eta_{p}^{2}$ = 0.28), with a significant main effect found for time (*p* = 0.01, 95%CI: −2.88 to −0.35). Specifically, in participants with lower baseline inter-limb asymmetry, significant (*d* = 1.27; *p* = 0.009; 95%CI: −2.82 to −0.41) differences emerged between PRE (34.8 ± 1.2 cm) and POST (33.2 ± 1.3 cm) evaluation. No significant interactions were detected.

Means, SD, contrast, 95% CI, and interaction effects of all variables are shown in Table 2.

## 4. Discussion

This study examined the acute effects of a single MTT session on unilateral and vertical jump performance and inter-limb asymmetry. Our findings showed a significant interaction between time (PRE vs. POST) and group (participants with higher vs. participants with lower baseline inter-limb asymmetry), whereby participants with higher baseline inter-limb asymmetry levels demonstrated a decrease in NSI (–4.0%) after MTT, whereas those with lower baseline inter-limb asymmetry levels showed a small, non-significant increase (4.6%), which remained below the critical cut-off. Although we hypothesized that asymmetry would remain stable in participants with lower baseline inter-limb asymmetry, the small increase observed following MTT should not be interpreted as a negative or clinically relevant outcome. Rather, it likely reflects a transient acute response to the training stimulus, potentially driven by post-session neuromuscular fatigue or short-term compensatory motor strategies adopted during post-intervention testing. Regarding 6MTH performance, participants completed the test faster after MTT, regardless of the limb used and the group, indicating an acute improvement in lower-limb functional performance.

Repetitive jumping on a mini-trampoline involves stretch–shortening cycle actions comparable to traditional plyometric training, but with reduced mechanical load due to the compliant surface [15,33]. Previous studies [18,27] have shown that MTT can enhance balance and leg stiffness, supporting its potential application in both preventive and rehabilitative contexts. Specifically, Di Rocco et al. [27] showed that, despite the elastic and compliant nature of the mini-trampoline surface, a single session induced a reduction in reactive power, interpreted as an acute neuromuscular fatigue response. In contrast, leg stiffness did not change, indicating that MTT might exert a protective effect on leg stiffness, preventing acute decreases that are commonly observed in other training modalities. Consistent with these findings, our results suggest that even a single session may help reduce inter-limb asymmetry in individuals with higher baseline values. The contrasting responses between groups can be explained by their different baseline asymmetry values. These group differences align with the concept of baseline dependency, in which individuals with greater initial asymmetry typically show larger acute improvements, a pattern also documented in previous plyometric training studies [18]. This reinforces that baseline asymmetry is a key factor influencing the magnitude of acute adaptations. Regarding vertical jump performance, CMJ performance decreased only in participants with lower baseline inter-limb asymmetry. This reduction may be attributed to acute neuromuscular fatigue associated with plyometric exercise, involving mechanical, neuromuscular, and metabolic factors [34]. The lack of decrease in the participants with higher baseline inter-limb asymmetry may indicate a more balanced force and power contribution from both limbs following the intervention. However, this remains speculative and cannot be confirmed without direct biomechanical measures. It should be noted that CMJ performance was not intended to assess inter-limb asymmetry per se, but rather to contextualize unilateral performance outcomes within a measure of overall bilateral neuromuscular function. The improvement observed in 6MTH performance, combined with the reduction or stability in CMJ performance, could also be related to the specific demands of the training. MTT primarily involves repeated bilateral jumps that more closely resemble the 6MTH task than the CMJ, possibly facilitating better neuromuscular activation patterns for hopping performance. A mild post-activation potentiation effect cannot be excluded, but should be considered only as a possible contributing factor, given that CMJ performance did not improve uniformly [35].

Overall, these findings indicate that MTT may acutely benefit individuals with higher baseline inter-limb asymmetry, while inducing a fatigue-related response in those with minimal asymmetry. This highlights how the same training stimulus may yield different short-term outcomes depending on initial functional status. From a healthcare perspective, these results have important implications as inter-limb asymmetry has been associated with increased musculoskeletal injury risk and reduced functional mobility [36]. In this context, the present findings support the potential role of MTT primarily in clinical and rehabilitative contexts, particularly for participants with higher baseline inter-limb asymmetry. The reduced impact forces of the mini-trampoline limit landing stress and may lower injury risk, making this modality suitable for populations with joint instability or balance deficits. Additionally, the accessibility and enjoyable nature of MTT may enhance long-term adherence to exercise interventions, which is essential for promoting sustained health benefits [37].

## 5. Conclusions

This study demonstrated that a single MTT session can acutely reduce inter-limb asymmetry, depending on baseline asymmetry values, during unilateral hopping without affecting CMJ performance in participants with higher baseline inter-limb asymmetry. Participants with higher baseline inter-limb asymmetry showed a reduction in NSI after MTT, compared to the participants with lower baseline inter-limb asymmetry, confirming that individuals with a higher level of asymmetry may achieve greater benefits in reducing performance imbalance during the 6MTH. From a practical perspective, mini-trampoline exercise represents an accessible and safe intervention that may be particularly beneficial within clinical and rehabilitative programs aimed at reducing functional inter-limb asymmetry in individuals with higher baseline asymmetry. Future research should investigate the long-term effects of MTT and evaluate its applicability in several populations, including sedentary adults, older individuals, and participants with higher baseline inter-limb asymmetry. Additionally, the use of automated measurement tools, such as timing gates or photocells, should be considered to enhance accuracy and reduce potential measurement error. Confirming its effectiveness across different settings could establish MTT as a valuable non-pharmacological intervention to improve functional capacity, prevent injury, and promote health and well-being across the lifespan.

## 6. Limitations

This study has several limitations that must be acknowledged when interpreting the findings. These include a relatively small sample size restricted to healthy young adults, the short term evaluation, and the absence of a non-exercise control group, which limits generalizability to clinical or older populations. Although participants with lower baseline inter-limb asymmetry were included as a reference comparison group to explore differential acute responses to MTT, this approach does not fully account for potential learning, fatigue, or time-related effects. Additionally, the study examined the short-term effects of a single session of MTT. Consequently, the present findings are specific to the acute response to exercise, whereas potential long-term effects necessitate further investigations. Using the 6MTH test to assess asymmetry may not capture all aspects of functional imbalance. Therefore, additional tests such as single hop, triple hop, and crossover hop could be considered for inclusion in future studies to better capture functional asymmetry. In addition, the lack of detailed biomechanical data (e.g., limb-specific force output or force symmetry during jumping tasks) limits the ability to explain the neuromuscular mechanisms underlying the preserved CMJ performance observed in participants with higher baseline inter-limb asymmetry.

## Figures and Tables

**Table 1:** Mean values and standard deviations of the participants’ characteristics.

	Participants with Lower Baseline Inter-Limb Asymmetry	Participants with Higher Baseline Inter-Limb Asymmetry	Total
N	16	12	28
Age (years)	24.3 ± 1.2	27.2 ± 2.8	25.6 ± 2.4
Body height (m)	1.59 ± 0.0	1.74 ± 0.1	1.65 ± 0.1
Body mass (kg)	56.6 ± 5.7	72.7 ± 11.9	63.5 ± 11.9
BMI (kg·m^−2^)	22.4 ± 2.3	24.0 ± 3.2	23.1 ± 2.5

N = Number; BMI = Body Mass Index.

**Table 2:** Means, standard deviations (SD), contrast, 95% confidence intervals (95% CI), and interaction effects of the 6 m Timed Hop Test (6MTH), Normalized Symmetry Index, and Countermovement Jump Test before and after the mini-trampoline training session.

		PRE	POST		Interactions
		Mean ± SD	Mean ± SD	Contrast (95%CI)	Time × Group
Participants with Higher Baseline Inter-Limb Asymmetry (n = 12)	6MTH in Dominant limb (s)	2.37 ± 0.59	2.37 ± 0.49	0.06 (−0.25 to 0.11)	*p* = 0.46
	6MTH in Non-dominant limb (s)	2.54 ± 0.39	2.33 ± 0.32	0.21 (−0.41 to −0.01)	*p* = 0.03
	Normalized Symmetry Index (%)	15.38 ± 3.81	11.38 ± 3.24	4.00 (−7.92 to −0.07)	*p* = 0.04
	Countermovement Jump (cm)	36.24 ± 3.24	35.17 ± 2.74	1.07 (−2.46 to 0.32)	*p* = 0.13
		PRE	POST		Interactions
		Mean ± SD	Mean ± SD	Contrast (95%CI)	Time × Group
Participants with Lower Baseline Inter-Limb Asymmetry (n = 16)	6MTH in Dominant limb (s)	2.53 ± 0.47	2.42 ± 0.44	0.11 (−0.27 to 0.04)	*p* = 0.16
	6MTH in Non-dominant limb (s)	2.48 ± 0.44	2.42 ± 0.52	0.06 (−0.23 to 0.11)	*p* = 0.46
	Normalized Symmetry Index (%)	5.02 ± 2.72	9.69 ± 6.12	4.66 (1.26 to 8.06)	*p* = 0.007
	Countermovement Jump (cm)	34.88 ± 4.93	33.26 ± 5.53	1.61 (−2.82 to −0.41)	*p* = 0.009

## Data Availability

The original data presented in the study are openly available at https://github.com/ccortis/DataHop.git.

## Supplementary Materials

The following supporting information can be downloaded at https://www.mdpi.com/article/10.3390/healthcare14020160/s1. Figure S1. Distribution of 6 m Timed Hop Test performance before (PRE) and after (POST) the mini-trampoline intervention for the dominant and non-dominant limb. The 6MTH performance was significantly (*p* = 0.01) different between PRE and POST MTT session regardless of group and limb. Figure S2. Distribution of the Normalized Symmetry Index before (PRE) and after (POST) the mini-trampoline intervention in both groups. * Significantly (*p* = 0.01) different between PRE and POST in both groups. # PRE values significantly (*p* < 0.001) different among groups; thus, the difference did not persist in the POST evaluation

## Abbreviations

The following abbreviations are used in this manuscript:

MTT	Mini-Trampoline Training
6MTH	6 m Timed Hop
CMJ	Countermovement Jump
NSI	Normalized Symmetry Index
N	Number
BMI	Body Mass Index
SD	Standard Deviation
CI	Confidence Interval
dl	Dominant Limb
ndl	Non-Dominant Limb
