# Supplementary material for: Acute Effects of a Mini-Trampoline Training Session for Improving Normalized Symmetry Index in Participants with Higher Baseline Inter-Limb Asymmetry
Source: Healthcare (Basel). 2026 Jan 8;14(2):160. doi: 10.3390/healthcare14020160 (PMC12841054; doi:10.3390/healthcare14020160)
Supplement: Supplementary file 1 [file healthcare-14-00160-s001.zip › healthcare-4067435-supplementary.pdf]

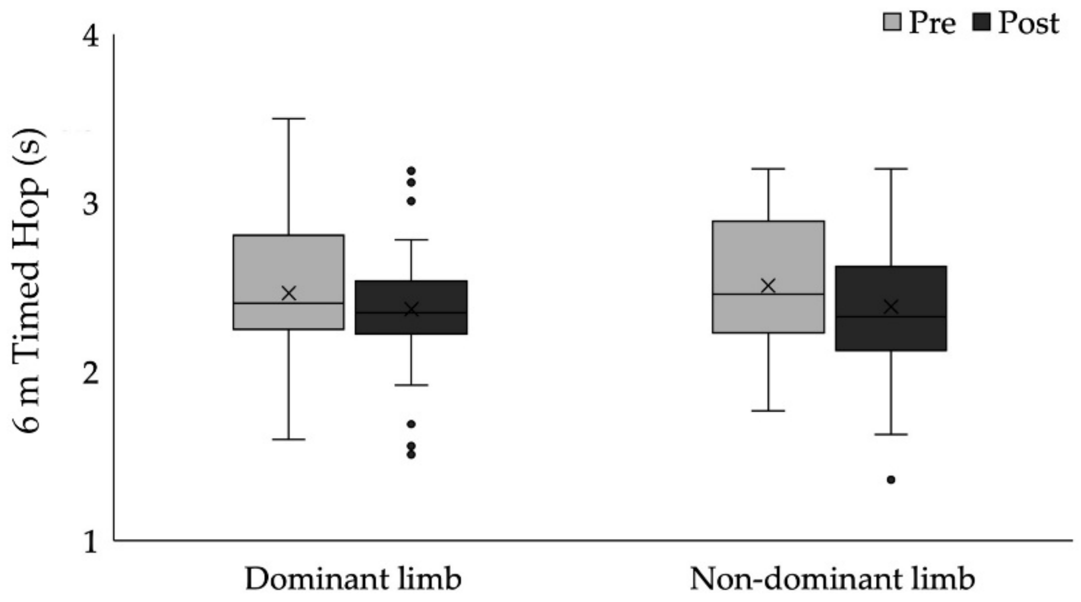

Figure S1. Distribution of 6 m Timed Hop Test performance before (PRE) and after (POST) mini-trampoline intervention for dominant and non-dominant limb. The 6MTH performance was significantly (p = 0.01) different between PRE and POST MTT session regardless of group and limb.

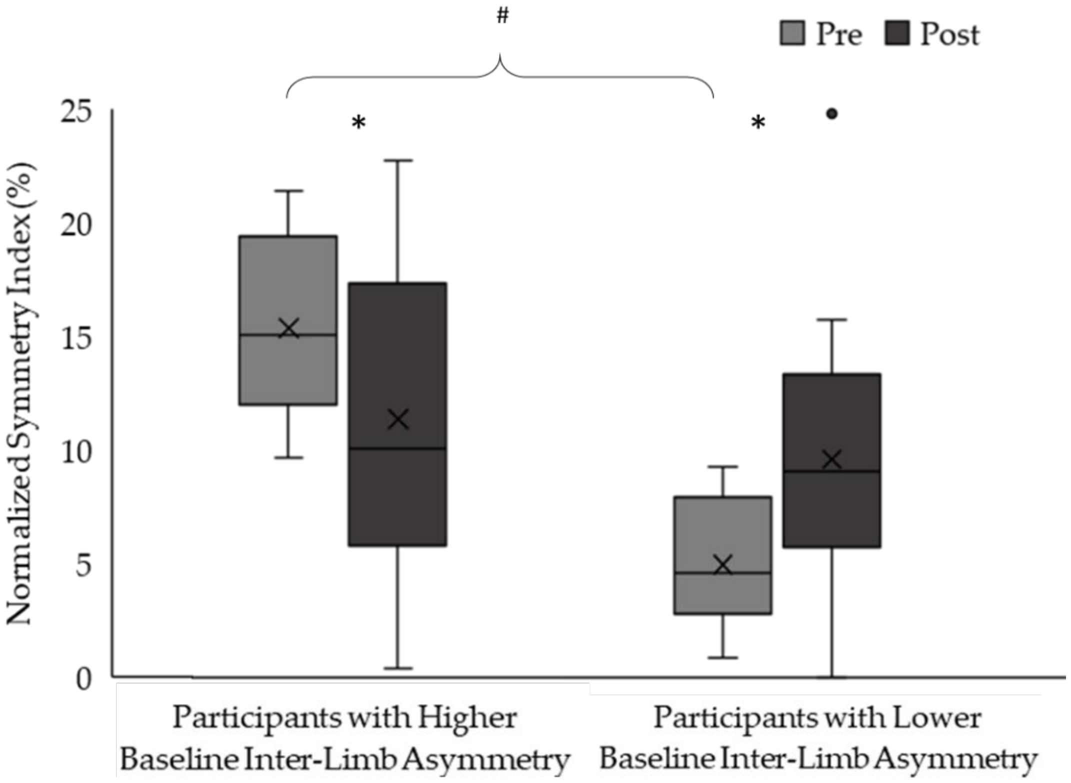

Figure S2. Distribution of Normalized Symmetry Index before (PRE) and after (POST) mini-trampoline intervention in both groups. * Significantly (p = 0.01) different between PRE and POST in both groups. # PRE values significantly (p < 0.001) different among groups, thus, the difference did not persist in POST evaluation.
